# Folding the unfoldable 2: using AlphaFold and ESMFold to explore spurious proteins

**DOI:** 10.1093/bioadv/vbag160

**Published:** 2026-06-10

**Authors:** Ailsa K Orr, Alex Bateman

**Affiliations:** European Molecular Biology Laboratory, European Bioinformatics Institute (EMBL-EBI), Hinxton, CB10 1SD, UK; European Molecular Biology Laboratory, European Bioinformatics Institute (EMBL-EBI), Hinxton, CB10 1SD, UK

## Abstract

**Motivation:**

Spurious protein sequences, resulting from gene prediction errors, theoretically should not yield folded structures. AlphaFold2 was previously shown to predict short spurious sequences with high pLDDT scores and was therefore unlikely to distinguish between real proteins and spurious proteins which are usually short. We evaluate whether newer structure prediction methods (ESMFold and AlphaFold3) similarly predict short sequences with high pLDDT or if they better discriminate between spurious and real proteins.

**Results:**

All three structure prediction methods (ESMFold, AlphaFold2, and AlphaFold3) predict short spurious sequences from AntiFam with unexpectedly high pLDDT scores, however the discrimination between spurious and real proteins improves beyond 100 amino acids. By analysing sequences with disparate pTM and pLDDT scores, we identified two potentially novel spurious shadow ORFs in Swiss-Prot and one potentially non-spurious AntiFam entry. Using the structure prediction scores, we developed a Gaussian Process Model and evaluated its performance on AlphaFold DB, identifying potential spurious proteins at scale. While limited on its own, this model can increase confidence in spurious protein identification when combined with other methods.

**Availability and implementation:**

Structure predictions are available at https://doi.org/10.5281/zenodo.20426908. Model implementation and figure generation code are available at https://github.com/0rra/fold_unfold2.

## 1 Introduction

Spurious proteins are the result of errors in protein-coding gene prediction. These erroneous entries arise from various sources, including non-coding RNA genes misidentified as protein coding (Tripp *et al.* 2011), genome contamination from repetitive elements (Breitwieser *et al.* 2019), or misidentification due to protein coding genes on the opposite strand (shadow ORFs), (Eberhardt *et al.* 2012, Trimble *et al.* 2012) or overlapping reading frames (Veloso *et al.* 2005).

As protein sequence databases continue to expand, the number of spurious proteins also increases. Only a small fraction of protein sequences is experimental verified or manually curated, the vast majority are automatically annotated. While an overall robust method, automatic gene prediction has a predicted error rate of 1–2% for bacterial genomes, which means potentially 2 to 5 million protein sequences in UniProtKB could be spurious.

AntiFam (Eberhardt *et al.* 2012) is a collection of profile hidden Markov models (HMMs) representing sequences believed to be spurious. It serves as a quality control tool for protein databases such as UniProtKB. With the release of version 8.0, AntiFam has grown to include 278 entries. AntiFam’s overall coverage of spurious proteins is likely to be low, but it is a useful training set for the development of more general methods to detect spurious proteins.

Previously AntiFam was used to evaluate AlphaFold2 (Jumper *et al.* 2021), and to investigate whether AlphaFold2 would assign globular structures to known spurious sequences (Monzon *et al.* 2022). That investigation revealed an important limitation: AlphaFold2 assigned high confidence scores (measured by predicted local distance difference test, pLDDT) to short AntiFam sequences, making them indistinguishable from real UniProtKB/Swiss-Prot proteins. This same effect was also found for randomly generated sequences. Additionally, one AntiFam entry (AntiFam: ANF00096, since removed) was identified as a false positive entry, containing real proteins rather than spurious ones.

Recent advances in protein structure prediction have introduced new methodologies: ESMFold (Lin *et al.* 2023), which employs a protein language model approach, removing the need for the time-consuming step of multiple sequence alignment creation, and AlphaFold3 (Abramson *et al.* 2024), which uses a generative diffusion approach distinct from AlphaFold2's neural network-based architecture. These methodological differences provide an opportunity to investigate whether these newer tools can better distinguish between spurious and real protein sequences.

In this study, we aim to:

1. Evaluate whether AlphaFold3 and ESMFold predict high confidence structures for short spurious sequences, as previously observed with AlphaFold2
2. Identify any false positive AntiFam entries
3. Investigate whether structure prediction confidence scores can be used to train a machine learning model which distinguishes between real and spurious proteins

We assess performance using confidence metrics including pLDDT (which measures local structural quality on a scale of 0–100) and predicted TM-score (pTM, which indicates the predicted global structural similarity to the native structure). In addition, an important distinction must be made between spurious proteins and intrinsically disordered proteins (IDPs). While both classes may exhibit low structural prediction confidences, IDPs are genuine functional proteins (Ruff and Pappu 2021). In order to evaluate how structure prediction methods perform, we aimed to avoid including IDPs in our real protein controls, by excluding proteins with a mean IUPred2a score above 0.5.

## 2 Methods

We used three types of input sequences for protein structure predictions: AntiFam sequences, randomly generated sequences, and UniProtKB/Swiss-Prot sequences (referred to as AntiFam, Random, and Swiss-Prot sequences, respectively).

AntiFam sequences were selected from AntiFam release 8.0, which includes 278 entries. Up to three sequences were selected from each AntiFam entry, with all sequences selected from smaller families of less than three sequences. Any gap characters within the sequences were removed, resulting in 763 AntiFam sequences, with lengths ranging from 16 to 886.

To compare prediction performance between spurious and real proteins, we selected sequences from UniProtKB/Swiss-Prot (version release 2025_03) and generated random sequences, with sample sizes and length bins defined by the distribution of AntiFam seed sequences across 20-residue intervals spanning 1–200 amino acids. Candidate sequences were filtered to remove (i) sequences flagged as fragments by UniProt and (ii) those with an average IUPred2A score above 0.5 to exclude disordered protein sequences (Mészáros *et al.* 2018, Erdős and Dosztányi 2020), and (iii) sequences with any AntiFam hits, to avoid spurious proteins in the real-protein set. From the remaining sequences within the 1–200 residue range, sequences were binned into 20-residue intervals and sampled from each bin to match the corresponding AntiFam bin count, totalling 644 Swiss-Prot sequences. Random sequences were also generated matching the AntiFam bin counts at 20-residue intervals within 1–200 residue range, with the amino acid composition weighting from Swiss-Prot, totalling 644 random sequences.

We ran structure predictions within a NextFlow pipeline, capable of performing AlphaFold2 (ColabFold 2.3.6), AlphaFold3 (commit 7f6edd04), and ESMFold [(fair-esm) version 2.0.0] predictions. The NextFlow pipeline and code used for plots can be found on https://github.com/0rra/fold_unfold2. The prediction confidence scores, mean pLDDT, pTM, and PAE (available for AlphaFold2 and AlphaFold3), for each structure prediction were recorded.

Contact order was computed from the predicted structures as a measure of structural complexity. For each predicted structure Cɑ coordinates were extracted from PDB or mmCIF files and residue pairs were defined as contacts if their Cɑ-Cɑ distance fell below 8 Å, and were separated by at least two residues in the sequence. Contact order was calculated as in Plaxco *et al.* (1998).

We trained a Gaussian Process Classifier (GPC) model using the three features, length, mean pLDDT, and mean pTM, to classify structures as spurious or real. Model training and testing metrics were calculated using Python 3.13, scikit-learn 1.6.1.

For the training data set, we used one sequence per AntiFam entry, excluding ANF00264, giving a total of 277 sequences. We matched this set with 277 bacterial Swiss-Prot sequences. To improve the model’s ability to distinguish short sequences, only sequences with a length less than or equal to 100 amino acids were used for training. To avoid data leakage, all bacterial sequences in Swiss-Prot were clustered with MMseqs2 (Mirdita *et al.* 2019), with coverage 90% and minimum identity 30%. For the training set we randomly sampled a single sequence from 277 clusters. Test sequences were selected randomly from the remaining clusters, to ensure cluster level separation between the training and test sequences.

Due to the limited number of AntiFams available, we generated AntiFam-like sequences to be used exclusively for model testing. These AntiFam-like sequences were made by translating protein-coding DNA of reference bacteria genomes in all possible frames and selecting sequences with stop and start codons to create spurious shadow ORFs and spurious frameshift protein sequences. The reference bacterial genomes were selected by randomly sampling 10 proteomes (Table 1) with BUSCO (Feron and Waterhouse 2022) score >95% and labelled “standard” by Complete Proteome Detector (CPD) (UniProt Consortium 2021), to select for complete and good quality proteomes. Ninety-six thousand eight hundred eighty synthetic sequences were produced and were then filtered to remove any which could possibly be real proteins, by filtering any with matches to Pfam, and also filtering any with sequence matches to bacterial reference proteomes (UniProtKB 2025_03) using Diamond protein sequence search (Buchfink *et al.* 2015). Sequences were also clustered using MMseqs2 as before, to keep only sequences from unique clusters. Ninety-one thousand eight hundred fifty-five AntiFam-like sequences passed these filtering steps, from which 150 per organism was sampled, normalising across length bins. The structures for the synthetic sequences were predicted using AlphaFold2, AlphaFold3, and ESMFold and the confidence scores were recorded.

**Table 1:** UniProtKB Proteome IDs of bacterial reference genomes sampled to generate AntiFam-like sequences.

UniProtKB Proteome ID	Organism
UP000656042	*Mangrovihabitans endophyticus*
UP000233375	*Niallia nealsonii*
UP000279194	*Streptococcus hillyeri*
UP000238169	*Caballeronia novacaledonica*
UP000260665	*Rhodoferax lacus*
UP000195781	[*Collinsella*] *massiliensis*
UP000001122	*Escherichia coli* O139: H28 (strain E24377A/ETEC)
UP000321303	*Halovibrio variabilis*
UP000008130	*Polymorphum gilvum* (strain LMG 25 793/CGMCC 1.9160/SL003B-26A1)
UP000190023	[Haemophilus] felis

Two test sets were constructed: a balanced set using 300 randomly sampled synthetic AntiFam-like sequences and 300 randomly sampled bacterial Swiss-Prot proteins (50% spurious set), and an unbalanced set containing 100 randomly sampled synthetic AntiFam-like sequences and 4900 randomly sampled bacterial Swiss-Prot proteins (2% spurious set), reflecting the estimated real-world prevalence of spurious sequences. The 300 Swiss-Prot sequences in the balanced set were downsampled from the 4900 in the unbalanced set, while the 100 AntiFam-like sequences in the unbalanced set were downsampled from the 300 in the balanced set, ensuring both test sets draw from a common pool. Swiss-Prot sequences used for testing were not length-restricted and were drawn from clusters absent from the training dataset. A summary of all datasets is provided in Table 2.

**Table 2:** Summary of GPC model training and testing datasets.

Dataset	Spurious (sequence number)	Spurious (sequence length)	Non-spurious (sequence number)	Non-spurious (sequence length)	Total
Training	277 (AntiFam, 1 per entry, excl. ANF00264)	16–886 (avg: 120.55)	277 (bacterial Swiss-Prot, length ≤ 100 aa)	17–100 (avg: 68)	554
Testing (50% spurious)	300 (synthetic AntiFam-like)	31–869 (avg: 177.75)	300 (bacterial Swiss-Prot)	49–968 (avg: 306.08)	600
Testing (2% spurious)	100 (synthetic AntiFam-like)	35–934 (avg: 186.25)	4900 (bacterial Swiss-Prot)	18–1545 (avg: 309.31)	5000

By default, GPC models use a threshold of 0.5. We optimised the classification threshold to maximise precision on the 2% test dataset before further application.

To assess how classifier performance scales with training data size, learning curves were generated by training the GPC model on stratified subsets of the full training data at fractions of 10%, 25%, 50%, 75%, and 100%. At each fraction, SwissProt vs Spurious proportions were preserved by sampling each sequence type independently. To account for variability in subset composition affecting the overall results we created five different random selections for each subset. Performance was summarised as the mean and standard deviation across the five random selections. Each model was evaluated on both the balanced (50% spurious) and unbalanced (2% spurious) test sets at each fraction, across four metrics: AUC, accuracy, average precision, and recall at 100% precision.

To assess classifier robustness and estimate performance variability, a resampling evaluation was performed by repeatedly drawing test sets from pooled held-out sequences. Two compositions were evaluated across 100 draws each: 50% spurious (100 AntiFam-like sequences and 100 Swiss-Prot sequences per draw) and 2% spurious (20 AntiFam-like sequences and 980 Swiss-Prot sequences per draw). For each draw, all GPC models were evaluated with the same sequences. Performance was recorded per draw across four metrics (AUC, average precision, accuracy, and recall at 100% precision), and summarised as the mean and 95% interval (2.5th–97.5th percentile) across draws.

Since AlphaFoldDB does not provide pTM scores directly, we derived an approximate pTM from the PAE matrix of each structure prediction (PAE-derived pTM). This approach was justified by strong agreement between PAE-derived pTM and ColabFold pTM scores (R^2^ > 0.9; Fig. S1). To apply the model to bacterial proteins with AlphaFold2 structures in AlphaFold DB, we extracted the requisite confidence scores, mean pLDDT and calculated an PAE-derived pTM from the PAE matrix of each protein structure using the following calculation:


$$p_{\text{TM}}=\underset{i}{\max}\frac{1}{N}\sum_{j=1}^{N}\frac{1}{1+{\left(\frac{{\text{PAE}}_{\mathrm{ij}}}{d_{0}}\right)}^{2}}$$

where the distance scaling factor $d_{0}$ (Zhang and Skolnick 2005) is defined as in AlphaFold2 [Supplementary equation 33 in Jumper *et al.* (2021)]:


$$d_{0}(N)=1.24\sqrt[{3}]{\max\left(N,\;19\right)-15}-1.8$$

N is the number of residues and ${\text{PAE}}_{\mathrm{ij}}$ is the predicted aligned error between residues i and j. The model, designated p-AF2-GPC where p stands for PAE-derived pTM, was trained using the same dataset of AntiFam and Swiss-Prot sequences used for the previous AlphaFold2 GPC model (AF2-GPC).

Only bacterial proteins which were present in both AlphaFold DB v6 and UniProt 2025_03 were used.

## 3 Results

### 3.1 Investigation of structure prediction method confidence scores

**Figure 1:**
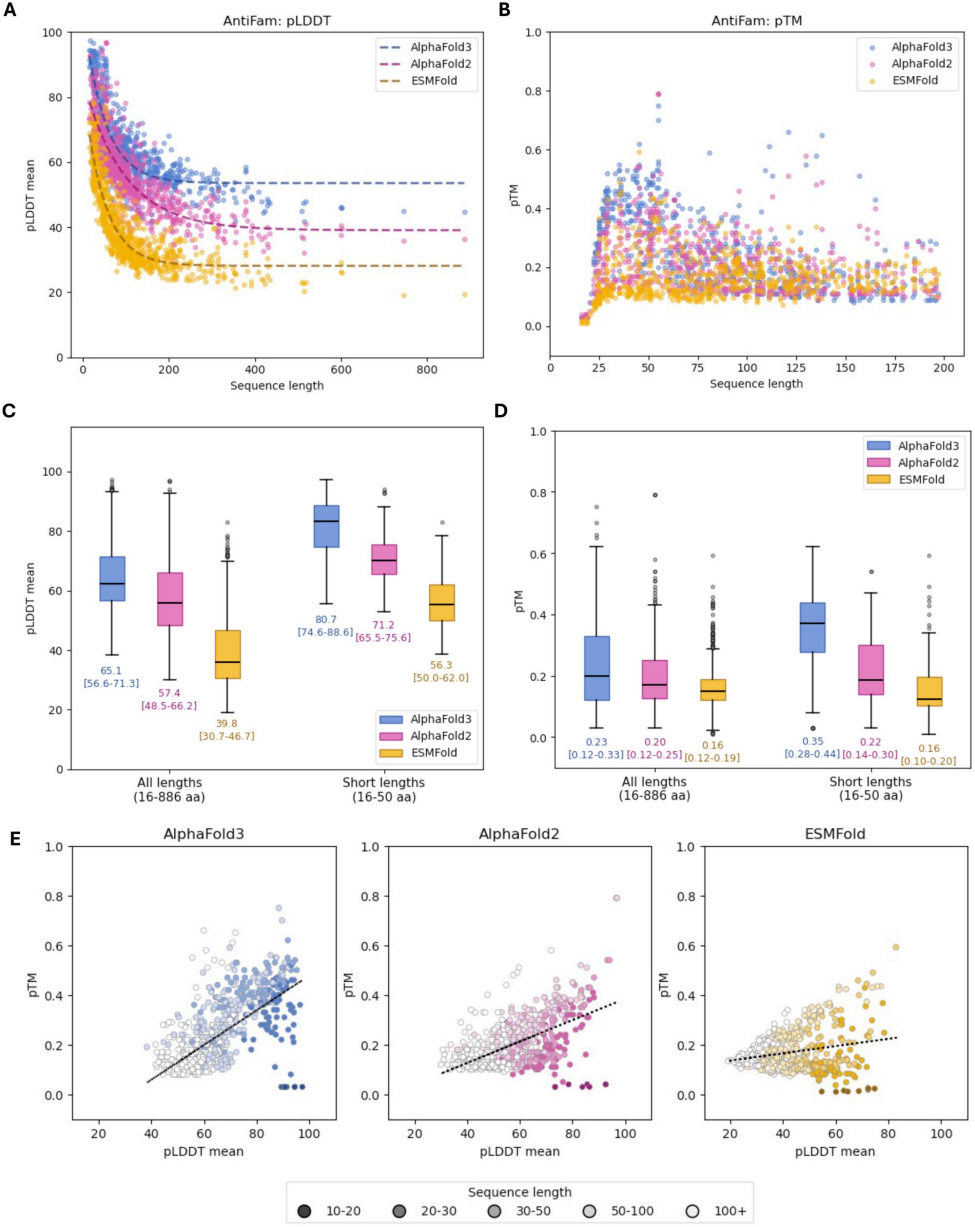
Confidence metrics for structures predicted from AntiFam sequences by ESMFold, AlphaFold2, and AlphaFold3. (A, B) Mean pLDDT scores and pTM by sequence length across all prediction methods. (C, D) Mean pLDDT score distributions per prediction method for all lengths vs short sequences (≤50), annotated with mean average score and IQR. (E) pTM versus mean pLDDT for each prediction method, coloured by sequence length.

We first examined whether ESMFold and AlphaFold3, like AlphaFold2 also produced surprisingly high pLDDT values for short spurious sequences (Fig. 1A). Structure predictions are considered to have high confidence (pLDDT >90), medium confidence (70–90), low confidence (50–70) and very low confidence (<50). pTM scores above 0.5 indicate confident global fold predictions.

All three methods predicted structures with higher mean pLDDT scores for shorter sequences. AntiFam sequence lengths average at 120 and range from 16 to 886 amino acids. The average mean pLDDT was highest for AlphaFold3 (65.15), followed by AlphaFold2 (57.36), and ESMFold (39.82). For short sequences (≤50 amino acids), all three methods predicted notably higher confidence scores, with mean pLDDT values of 80.74, 71.18, and 56.3 for AlphaFold3, AlphaFold2 and ESMFold respectively, an increase over the mean of 24% for both AlphaFold3 and AlphaFold2, and 41% for ESMFold. However, despite ESMFold showing the largest relative increase for short sequences, it still predicted with lower confidence overall. The range of mean pLDDT values was most similar between AlphaFold2 (30.0–96.9) and AlphaFold3 (38.3–97.4), while ESMFold predicted lower confidence scores overall (19.1–82.9).

For pTM versus length (Fig. 1B), all three methods show a clustering of sequences with low pTM (under 0.05), for protein lengths below 25. AlphaFold2 and AlphaFold3 predicted some protein structures with high pTM scores (over 0.5) compared to equivalent-length sequences, as shown by the outlying points in Fig. 1B. Across all lengths, pTM distributions overlapped between methods with the overall average pTM scores being 0.23, 0.2, and 0.16 for AlphaFold3, AlphaFold2 and ESMFold. For short sequences AlphaFold3 shows a larger increase in average pTM to 0.35, while AlphaFold2 (0.22) and ESMFold (0.16) show little to no increase in average pTM. This suggests that AlphaFold3 inflates both pLDDT and pTM for shorter spurious sequences.

When examining the correlation between mean pLDDT and pTM, ESMFold showed the weakest linear correlation (Fig. 1E). AlphaFold3 showed the stronger correlation between mean pLDDT and pTM (0.42), while AlphaFold2 showed a weaker correlation (0.31). For all three methods, it was also observed that shorter sequences (under 20 residues) had consistently low pTM (under 0.1), but high pLDDT scores, with AlphaFold3 predicting these short AntiFam sequences with very high pLDDT scores, greater than 80.

**Figure 2:**
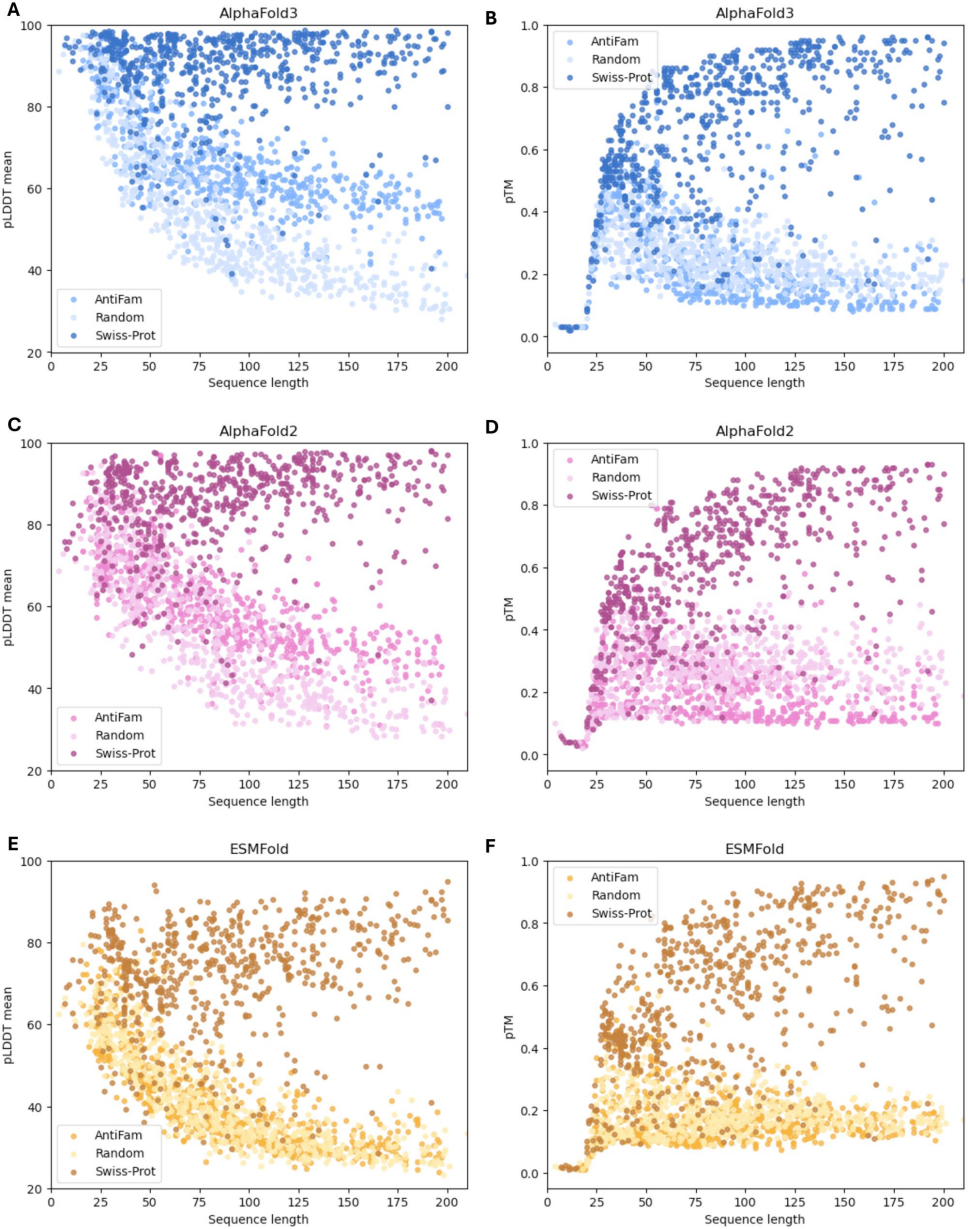
Comparison of prediction confidence across structure prediction methods, using AntiFam, Random and Swiss-Prot sequences. Mean pLDDT across sequence lengths for (A) AlphaFold3, (B) AlphaFold2, and (C) ESMFold. pTM across sequence lengths for (D) AlphaFold3, (E) AlphaFold2, and (F) ESMFold.

Comparing AntiFam, Random and Swiss-Prot structure predictions, Swiss-Prot proteins generally were predicted with higher confidence across all three methods (Fig. 2). Mean pLDDT for Swiss-Prot predictions was 89.3, 85.5, and 72,2 for AlphaFold3, AlphaFold2, and ESMFold, which is substantially higher than the average pLDDT scores for both AntiFam predictions and Random predictions (55.1, 54, 42). The same trend was observed for pTM scores where Swiss-Prot average pTM scores were 0.67, 0.61, and 0.56 for AlphaFold3, AlphaFold2, and ESMFold, which is roughly three times higher compared to AntiFam pTM scores across the three methods.

Both AlphaFold methods predicted AntiFam structures with higher confidence compared to Random structures, particularly AlphaFold3. However, for pTM scores, Random structures were predicted with slightly higher scores compared to AntiFam by AlphaFold2 and AlphaFold3, while ESMFold showed little difference.

Across all three methods the majority of Swiss-Prot were predicted with confident mean pLDDT (>70) across sequence lengths (94.1% AlphaFold3, 90.8% AlphaFold2, and 65.7% ESMFold Swiss-Prot structure predictions), in contrast to the length-dependent declines observed for both AntiFam and Random predictions. For sequences longer than 50 residues, the vast majority were also predicted with confident pTM scores (>0.5) (85.0% AlphaFold3, 80.8% AlphaFold2, and 73.6% ESMFold).

To quantify the degree of separation between sequence types across lengths, mean pLDDT scores were binned into 20-residue intervals and the separation between AntiFam and Swiss-Prot distributions were assessed per bin using the overlapping coefficient and AUROC (Fig. S2). The overlapping coefficient (OC) measures the proportion of shared probability between the two distributions, the regions where both groups are likely to occur. The OC was calculated by fitting a kernel density estimate (KDE) to each group’s pLDDT values per bin, and integrating the minimum of the two curves across the range of pLDDT values, giving a measure of 0 (no overlap) to 1 (identical distributions). The AUROC was computed as the probability of a randomly drawn AntiFam sequence with scores lower than a randomly drawn Swiss-Prot sequence, ranging from 0.5 (no separation) and 0 (complete separation), to measure how consistently Swiss-Prot sequences receive higher scores than AntiFam sequences. Separation in pLDDT was weakest at shorter lengths, with substantial overlap throughout 0–60 aa bins across all three methods. From 60 aa onwards, separation increased progressively with AUROC falling below 0.1 for sequences longer than 100 aa for all three methods.

The full distribution of per-residue pLDDT scores across sequence types and length bins for AntiFam, Random, Swiss-Prot predictions across all methods is available in Fig. S3, and the full per-residue-pair PAE distributions for AlphaFold3 and AlphaFold2 is available in Fig. S4. The contact order of predicted structures across sequence types, reflecting the degree of long-range versus local contact formation, is shown in Fig. S5. All three structure prediction methods predict AntiFam structures with low contact order across sequence lengths.

A small subset of 33 Swiss-Prot proteins fell below the average AntiFam mean pLDDT score for each method (23 for AlphaFold3, 13 for AlphaFold2, and 23 for ESMFold). Of these, five were flagged by UniProt with caution labels and uncertain existence annotations. Four of these were yeast proteins, 3 of which were longer than 100 aa and flagged as dubious gene predictions unlikely to encode functional proteins (Q6B0Z2, P40490, and A0A023PXK2), and one (Q06057, 63 aa) was flagged for erroneous initiation. These proteins have already been identified and removed from the complete/reference *S. cerevisiae* S288c proteome set (Engel *et al.* 2014). Besides these yeast proteins, A4GYV5 (45 aa, *Populus trichocarpa*), a putative chloroplast protein, was also flagged as a possible pseudogene product.

Beyond these cautioned proteins, two additional proteins were identified which may represent previously unidentified spurious ORFs. O29555 (89 aa, *Archaeoglobus fulgidus*) has existence inferred from homology only and a blastx search of its DNA sequence returned only three hits, all to uncharacterised *Archaeoglobus* proteins in the −3 frame, raising the possibility this protein may be a spurious shadow ORF. Q99166 (91 aa, *Bacillus licheniformis*) has stronger spurious ORF evidence. This protein is classified as predicted existence only and blastx hits were exclusively in the negative frame of phage-related proteins across *Bacillus* species, suggesting xpaR7 resides in a prophage region. The low mean pLDDT scores from all three structure prediction methods (39.2, 43.2, 29.5) indicate it may be non-functional. These low-confidence outliers highlight the ability of structure prediction methods to identify potentially misannotated sequences within curated databases.

### 3.2 Identification of a potential false-positive AntiFam

**Figure 3:**
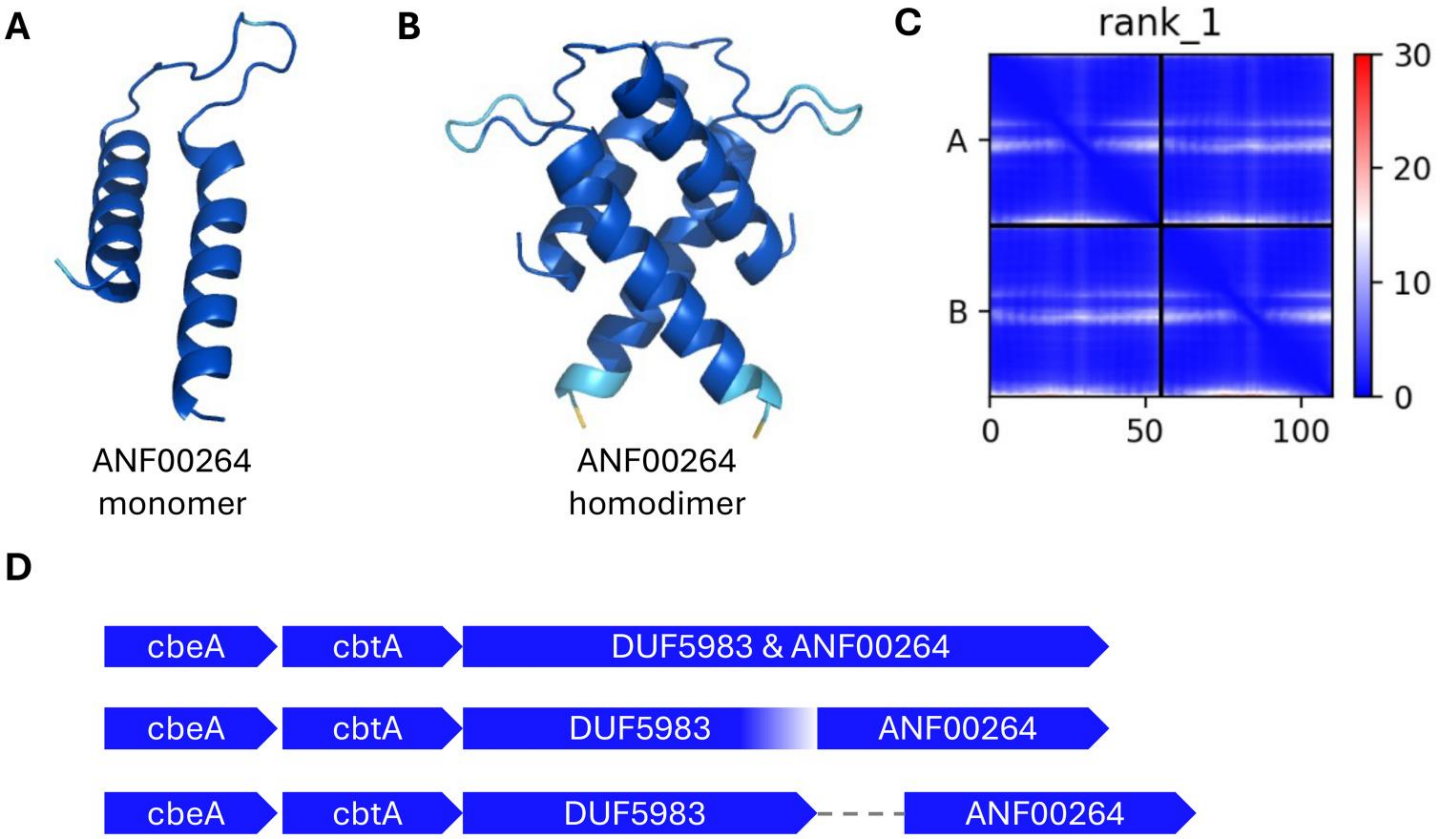
AlphaFold2 structure predictions of potential non-spurious AntiFam entry ANF00264. ANF00264 monomer, coloured by pLDDT (A). ANF00264 homodimer, also coloured by pLDDT (B). Predicted Aligned Error (PAE) plot of ANF00264 homodimer to show confidence of relative position of residues and domains within the structure (C). Local genomic contexts of ANF00264: DUF5983 and ANF00264 predicted as part of a single protein, incomplete C-terminus DUF5983 and ANF00264 predicted as separate proteins, and distinct DUF5983 and ANF00264 predicted proteins separated by a non-coding region (D).

We inspected the structures of AntiFam structures with the highest pTM and pLDDT for globular, protein-like structures. The AntiFam ANF00264 predictions had the highest pTM and were also one of the AntiFam predictions with high pLDDT which were not a single alpha-helix. We tested whether this protein might form a homodimer by generating a dimeric structure with AlphaFold2 (Fig. 3B). The PAE plot supported interactions between the two homodimeric subunits (Fig. 3C). The mean pLDDT was 94.8, the pTM was 0.881, and the ipTM was 0.87. No other AntiFam entries were found to have globular structure.

AntiFam describes ANF00264 as a set of spurious proteins derived from frameshifted phage proteins. Although frameshifts often indicate spuriousness, programmed frameshifts have been documented in several Bacteriophage proteins (Sipley *et al.* 1991, García *et al.* 2004, Xu *et al.* 2004), raising the possibility that ANF00264 could represent a genuine programmed frameshift protein.

A blastp search performed using the first seed sequence of ANF00264 returned a 100% match to a hypothetical protein (UniProtKB: A0A083ZZA7) found in *Serratia Sp.*, as well as more distant matches in other *Enterobacteriaceae* species. We examined the domains of this protein and found it had matches to ANF00264 as well as the Pfam domain, “Family of unknown function (DUF5983) C-terminal domain” (Pfam: PF19419), which is also described as likely phage derived (Paysan-Lafosse *et al.* 2025). Extending this search, we also identified a further 24 proteins containing both ANF00264 and DUF5983 matches. Among these proteins, eight are named as CP4-44 prophage proteins and four as Aec77 proteins. These proteins occur in multiple bacterial species including *Escherichia coli*, *Klebsiella pneumoniae*, and *Shigella flexneri*. Notably one of these proteins (UniProtKB: A0A447X825) also contained the CbtA toxin domain (Pfam: PF06755).

CbtA (Cytoskeleton binding toxin A) inhibits cell division by binding to cytoskeleton proteins FtsZ and MreB, ultimately causing cell death. CbtA functions with the antitoxin CbeA within a toxin-antitoxin (TA) system, where both genes are typically encoded adjacently within the same operon (Brown and Shaw 2003).

This finding of ANF00264 adjacent to a well-characterised operon, prompted us to examine whether this was an isolated case of a conserved feature in other bacteria with the CbtA system. We found that the proteins containing the ANF00264 and DUF5983 matches were consistently found to be encoded next to cbtA and cbeA genes.

Interestingly, ANF00264 did not always appear as part of a multidomain protein with a DUF5983 domain. Sometimes it was predicted as a separate protein, sometimes separated by a short non-coding region. We hypothesise that this difference reflects the programmed frameshift, where annotation tools may predict a fused or separate protein. This is also possible given assemblies where DUF5983 protein upstream of ANF00264 was annotated to have an incomplete C-terminus, resulting in two distinct predicted proteins.

The repeated association of ANF00264 with a functional operon, despite variation in annotation, suggests it is not a spurious sequence but instead may be a phage-derived domain or protein associated with the CbtA TA system.

### 3.3 Evaluation of Gaussian Process Classification (GPC) models trained with structure prediction results

To test whether prediction confidence scores could be used to systematically distinguish between real and spurious proteins, we trained three GPC models [AlphaFold2-GPC (AF2-GPC), AlphaFold3-GPC (AF3-GPC), and ESMFold-GPC (ESM-GPC)]. The models used three features, sequence length, and confidence scores mean pLDDT and pTM which were derived from each respective structure prediction method. Model precision was prioritised over recall, to minimise incorrectly classifying real proteins as spurious, and recall at 100% precision was used as the primary evaluation metric.

**Figure 4:**
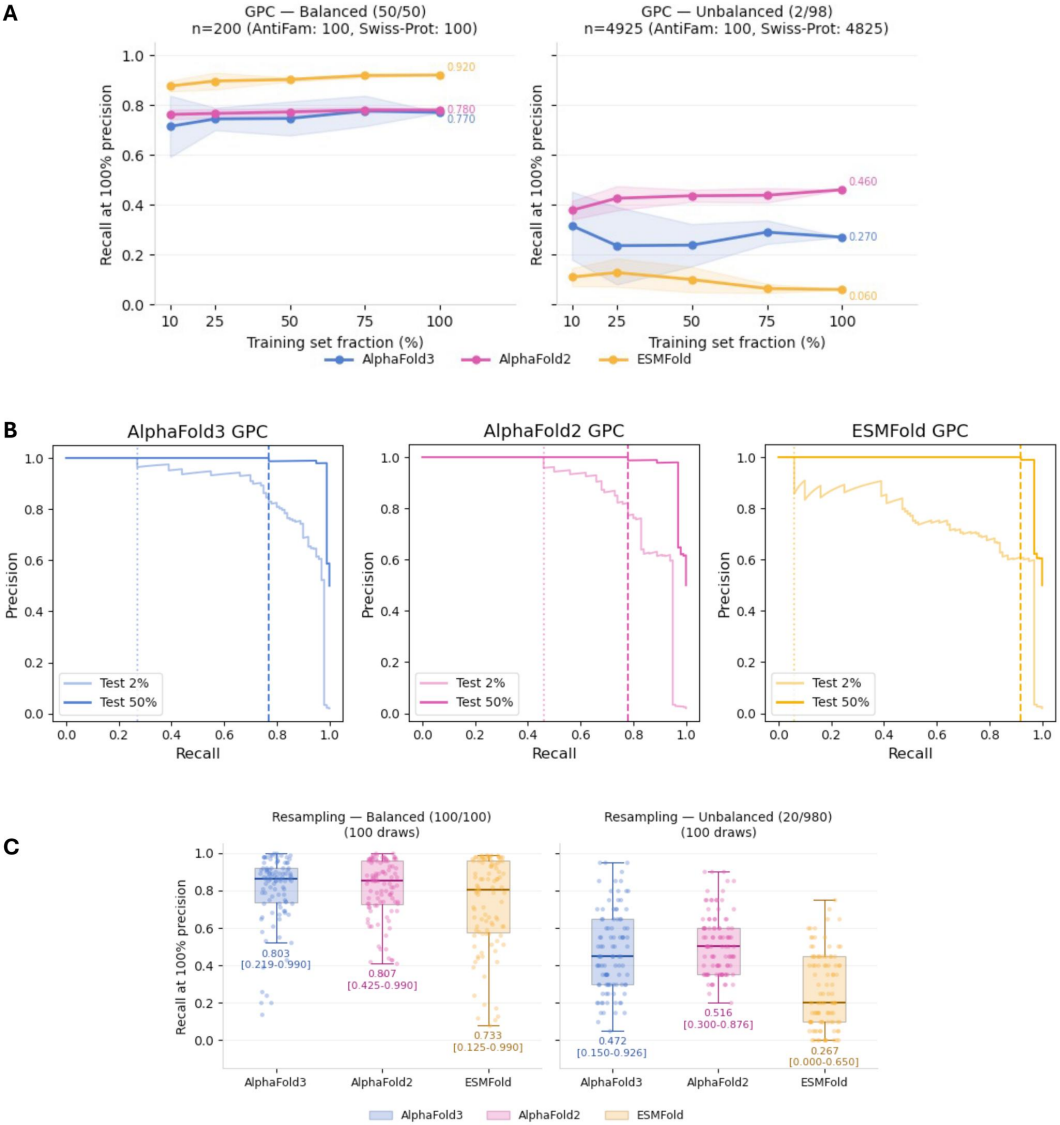
Models were tested on balanced datasets constructed from 50% synthetic AntiFam structure predictions and 50% Swiss-Prot structure predictions, and unbalanced datasets with 2% synthetic AntiFam structure predictions and Swiss-Prot structure predictions. (A) Learning curves across five training set fractions (10–100%), five random sets per fraction, lines show mean and shaded bands for ±1 SD. (B) Precision-recall curves for GPC models tested on 2% spurious test set shown by the darker lines and 50% spurious test set shown by lighter lines. Dashed lines mark maximum recall at 100% precision using the unbalanced test set, and dotted lines mark maximum recall at 100% precision using the balanced test set. (C) Bootstrap resampling evaluation (100 draws) under balanced and unbalanced datasets, darker line at median and boxes spanning IQR with whiskers at 1.5x IQR. Mean and 95th percentile intervals annotated below.

As the GPC models were trained on relatively small datasets (under 300 Swiss-Prot proteins and AntiFam proteins), learning curves were generated across five training set fractions (10–100%) to confirm the performance was not dependent on training set size (Fig. 4A). Across all three models, recall at 100% plateaued by the 100% training fraction when tested with both balanced and unbalanced datasets, indicating its performance was not limited by the available training data. Learning curves for additional performance metrics are available in Fig. S6.

We generated precision-recall curves for each model (Fig. 4B) to identify if the models maintained reasonable recall rates, if optimised for 100% precision. We evaluated the models using both a balanced test set and an unbalanced dataset, reflecting the occurrence of spurious sequences in protein databases. When tested on the balanced dataset (50% spurious), ESM-GPC demonstrated the best performance with 92% recall at 100% precision. AF2-GPC achieved 78% recall at 100% precision, closely followed by AF3-GPC with 77% recall at 100% precision. When tested on the unbalanced dataset (2% spurious), all models experienced a decrease in recall at 100% precision. The AF3-GPC recall at 100% precision decreased to 27%, ESM-GPC decreased greatly to 6% and AF2-GPC decreased to 46%. Summary performance metrics for all three models are shown in Table 3.

**Table 3:** Summary of model performance metrics for AlphaFold2-GPC, AlphaFold3-GPC and ESMFold-GPC.

GPC Model	Test set	AUC	Average precision	Accuracy	Recall at 100% precision
AlphaFold3	Balanced (50%)	0.99	0.993	0.79	0.77
AlphaFold3	Unbalanced (2%)	0.983	0.894	0.626	0.27
AlphaFold2	Balanced (50%)	0.98	0.986	0.86	0.78
AlphaFold2	Unbalanced (2%)	0.963	0.866	0.74	0.46
ESMFold	Balanced (50%)	0.981	0.988	0.88	0.92
ESMFold	Unbalanced (2%)	0.975	0.767	0.787	0.06

Bootstrap resampling (Fig. 4C) was used to assess variability in performance across different samples of the test data, to reflect the variability of real and spurious sequences in protein databases. Bootstrap resampling revealed AF3-GPC performance on the unbalanced set was higher than the single precision-recall curve suggested, as the mean recall was 47.2%, comparable to AF2-GPC with a mean recall of 51.6%. All models showed substantial variability across draws, showing sensitivity to the test sample, this variability increased when tested with the unbalanced samples. ESM-GPC performance was particularly unreliable on test sets where spurious proteins were realistically rare. Bootstrap resampling results for additional performance metrics are available in Fig. S7.

### 3.4 Applying classification model on AlphaFold DB

We also explored the possibility of applying the AF2-GPC on AlphaFold DB (Varadi *et al.* 2024), a large-scale database of structure predictions. As AlphaFold DB lacks pTM scores, we calculated PAE-derived pTM scores from the available PAE matrices (see Methods). Using PAE-derived pTM, mean pLDDT and length as features, we trained a model designated p-AF2-GPC on the same AntiFam and Swiss-Prot datasets used for AF2-GPC. When tested on the unbalanced test set, p-AF2-GPC achieved 32% recall at 100% precision, with a classification threshold of 0.957.

AlphaFold DB consists of sequences from two main sources: UniProtKB reviewed entries (Swiss-Prot), which represent manually curated, high-quality protein sequences and UniProtKB unreviewed entries (TrEMBL), which are computationally annotated and may contain errors or spurious sequences. We focused on bacterial proteins for two main reasons; our collection of spurious families (AntiFam) are largely from bacterial sequences, and eukaryote proteins more commonly exhibit disordered regions (Basile *et al.* 2019) which reduces structure prediction confidence, which may lead to false positive predictions. AlphaFold DB has 115 057 521 bacterial TrEMBL protein structure predictions, and 335 771 bacterial Swiss-Prot protein structure predictions.

Applying p-AF2-GPC on bacteria-only-AlphaFold DB returned 378 358 TrEMBL proteins (0.3% of bacterial TrEMBL proteins) and 105 Swiss-Prot proteins (0.03% of bacterial Swiss-Prot proteins) predicted as spurious. While the number of Swiss-Prot proteins is low, further investigation was performed to investigate if all 105 were indeed spurious proteins.

**Figure 5:**
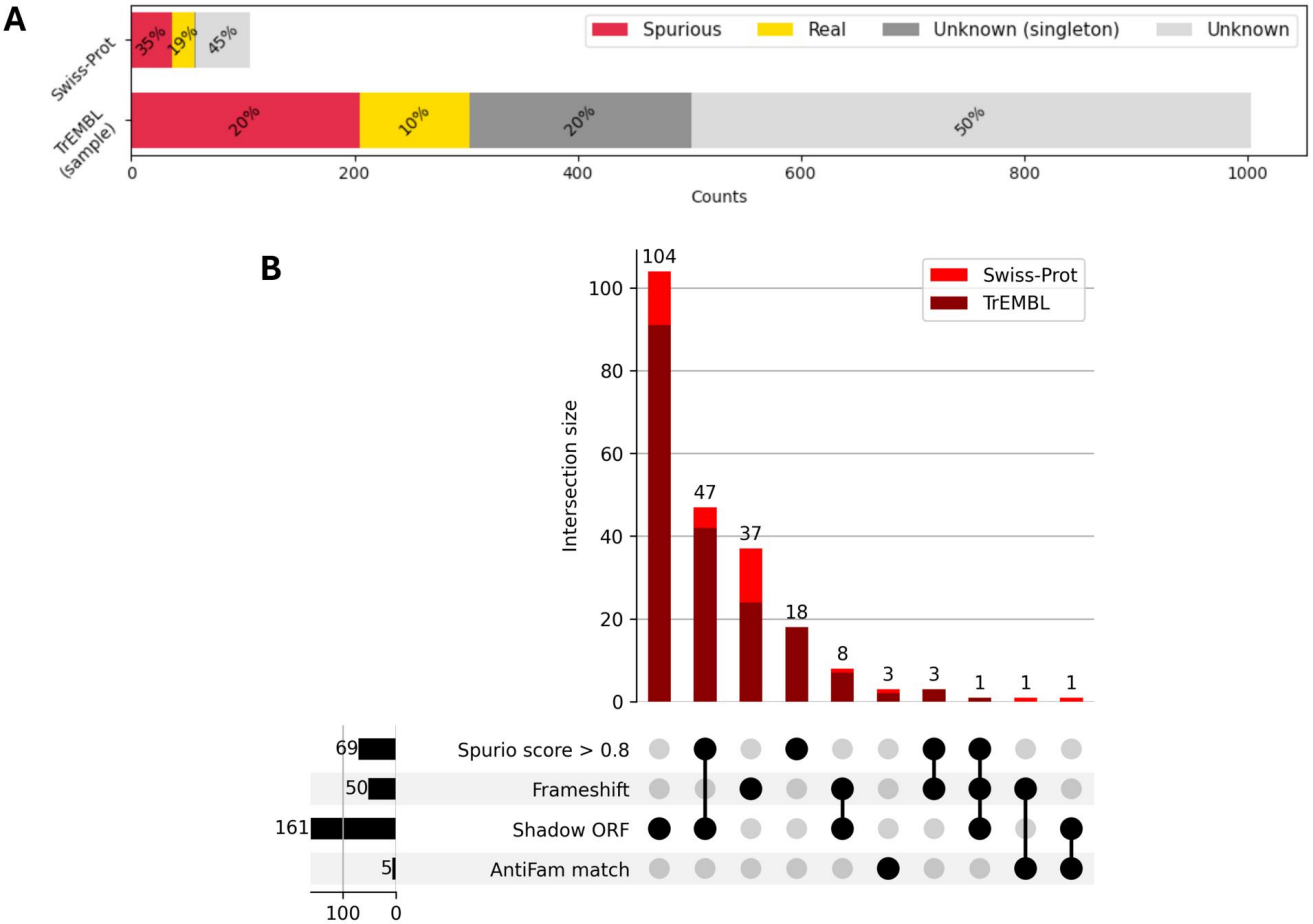
(A) Investigation results for proteins predicted as spurious by p-AF2-GPC: all spurious Swiss-Prot proteins and a random sample (1000) of predicted spurious TrEMBL proteins. Additional evidence was used to assess the proteins as spurious, real proteins (false positives), and unknown (insufficient evidence to confirm or reject prediction). Unknown proteins were subdivided into singleton proteins (potential orphan genes with few or no sequence matches). (B) UpSet plot showing the overlap of four additional spurious protein detection methods used to validate p-AF2-GPC predictions. Horizontal bars show total number proteins predicted as spurious by each method. Vertical bars show the number of proteins in each intersection and the stacks are coloured to distinguish Swiss-Prot (red) and TrEMBL (dark red).

The proteins predicted as spurious were assessed to be likely real if they have Pfam matches, or experimental evidence at the protein level. Protein evidence scores were obtained from the UniProtKB entry annotations for each protein. The scores scale from to 1–5 depending on strength of existence evidence where 1 is direct protein detection. Proteins were assessed to be likely spurious if they have AntiFam matches, score highly with tools such as Spurio (Höps *et al.* 2018), or are found to be frame mispredictions of well-known proteins (shadow ORF or frameshift sequences). The summary of these assessments is shown in Fig. 5.

Out of the 105 Swiss-Prot proteins p-AF2-GPC predicted as spurious, 20 were found to have at least one match to a Pfam family and or experimental evidence, and therefore were likely real proteins. A quarter of these were also classed as disordered by MobiDB consensus (Piovesan *et al.* 2025). Thirty-seven of the Swiss-Prot proteins were assessed to be likely spurious as they either had AntiFam matches, Spurio score >0.8, shadow ORF or frameshift evidence.

The TrEMBL proteins are harder to classify, as the large majority are not experimentally characterised. However much of TrEMBL is still covered by Pfam, 76% of proteins have a match to Pfam. We randomly sampled 1000 of the TrEMBL proteins predicted by the model to be spurious to gauge if there was a similar false positive prediction rate. From the sample we found 98 proteins with Pfam matches, and out of these real proteins, 36 were predicted to be intrinsically disordered. This left 700 unclassified proteins, 405 of which were predicted to be intrinsically disordered. Two hundred five proteins from the TrEMBL sample were assessed to be likely spurious, either having AntiFam matches, Spurio score >0.8, shadow ORF or frameshift evidence.

Among the proteins assessed as likely spurious, shadow ORF evidence was the most common indicator, with 104 proteins supported with this evidence alone. A further 47 proteins showed both shadow ORF and a high Spurio score (>0.8), these represent the highest-confidence spurious predictions in the dataset. These 47 proteins alongside additional candidates with high Spurio scores and frameshift evidence were compiled into a set of confident spurious predictions (https://github.com/0rra/fold_unfold2/blob/main/part3_gpc_testing/3_results_plotting/results/spurious_candidates.tsv).

Notably, only five proteins out of the assessed model predictions have AntiFam matches, three of which were from Swiss-Prot. This suggests that the majority of spurious proteins identified here are novel findings not previously captured by AntiFam. This highlights an advantage of p-AF2-GPC over established methods, as AntiFam is limited to known spurious families, missing novel ones, and while Spurio can detect a broader range, it is more intensive, less scalable and prone to predict transposon related proteins as spurious. Contrastingly, the GPC model requires only three structural features which allows it to scale across large structure databases to find proteins previously overlooked by past approaches.

## 4 Discussion

We found that ESMFold, AlphaFold2 and AlphaFold3 all predict short sequences with high pLDDT values, regardless of whether the sequences represent spurious or randomly generated sequences. As a result, significant separation in pLDDT between spurious and real sequences only becomes apparent when sequence length exceeds 100 amino acids.

A likely cause for the length bias could be there are fewer possible structural arrangements for shorter sequences to adopt, therefore can more easily be predicted, contributing to higher pLDDT values even when the underlying sequence may be spurious. Very short sequences under 30 amino acids, real and spurious, are also predicted with very low pTM scores, as well as high pLDDT. To further investigate this pattern in confidence scores for short sequences and the seemingly limited structural variation (e.g. alpha-helix structure), we can inspect the predicted structures of a larger pool of experimentally verified short proteins.

Out of the 644 randomly sampled proteins from UniProtKB/Swiss-Prot, at least five were found to be likely spurious proteins, which suggests Swiss-Prot may contain nearly 1% of spurious proteins (0.78%). However, this may be an overestimate as many of these proteins originate from the same organism strain, suggesting an error localised to a particular genome rather than a widespread error throughout Swiss-prot. The majority of spurious proteins found were approximately 100 amino acids or longer [with the exception of A4GYV5 (45 aa) and Q06057 (63 aa)], consistent with our finding that structure prediction confidence becomes more discriminative above this length.

We also observed that AlphaFold2 and AlphaFold3 differentiated Random sequences from AntiFam sequences, predicting Random sequences with lower confidence. This likely stems from their architectural design, as both methods use multiple sequence alignment (MSA), and the lower number of matches in the MSA for random sequences results in lower confidence predictions. AntiFam sequences may have higher coverage than random sequences due to matching with other spurious protein sequences remaining in sequence databases. ESMFold does not use an MSA, perhaps explaining why it does not differentiate between AntiFam and Random sequences.

While the GPC model has a non-zero false positive prediction rate, it can effectively enrich candidate spurious proteins, combined with additional methods such as Spurio. The sampled flagged TrEMBL proteins included 20% spurious sequences, compared to the estimated 1–2% in TrEMBL overall. While the model does misidentify some real proteins, only 10% of the flagged TrEMBL proteins had Pfam matches versus the 76% Pfam coverage of TrEMBL overall. This suggests the model successfully enriches spurious proteins in its output. The model could be used to quickly narrow down potential spurious proteins given structure prediction confidence scores, and be used in combination with more time-consuming methods to further validate and identify spurious proteins with greater confidence.

The remaining unexplained proteins without Pfam matches may represent genuine but uncharacterised proteins, sequences with few to no homologs (orphan genes) or spurious sequences which evade current detection methods. Limited homology may indicate either novelty i.e. de novo gene birth, or lack of evolutionary conservation as would be expected of spurious sequences. A similar issue remains with intrinsic disorder, as this property appears coincidentally with spurious protein sequences.

In conclusion, we have shown that all structure prediction methods tested predict short sequences with high pLDDT. This hampers our ability to use structure prediction metrics to identify spurious proteins which are often short. We present a Gaussian Process Classifier method which distinguishes real and spurious proteins imperfectly, but it is fast to apply and can be used as a pre-filtering step before using more computationally expensive methods to identify spurious proteins. The identification of spurious proteins remains an important but unsolved challenge in protein bioinformatics.

## Supplementary Material

vbag160_Supplementary_Data

## Data Availability

The structure predictions underlying this article are available at https://doi.org/10.5281/zenodo.20426908, and the structure prediction NextFlow pipeline and code used for figure generation can be found at https://github.com/0rra/fold_unfold2.
