# Supplementary material for: Folding the unfoldable 2: using AlphaFold and ESMFold to explore spurious proteins
Source: Bioinform Adv. 2026 Jun 10;6(1):vbag160. doi: 10.1093/bioadv/vbag160 (PMC13294457; doi:10.1093/bioadv/vbag160)
Supplement: vbag160_Supplementary_Data [file vbag160_supplementary_data.pdf]

Supplementary

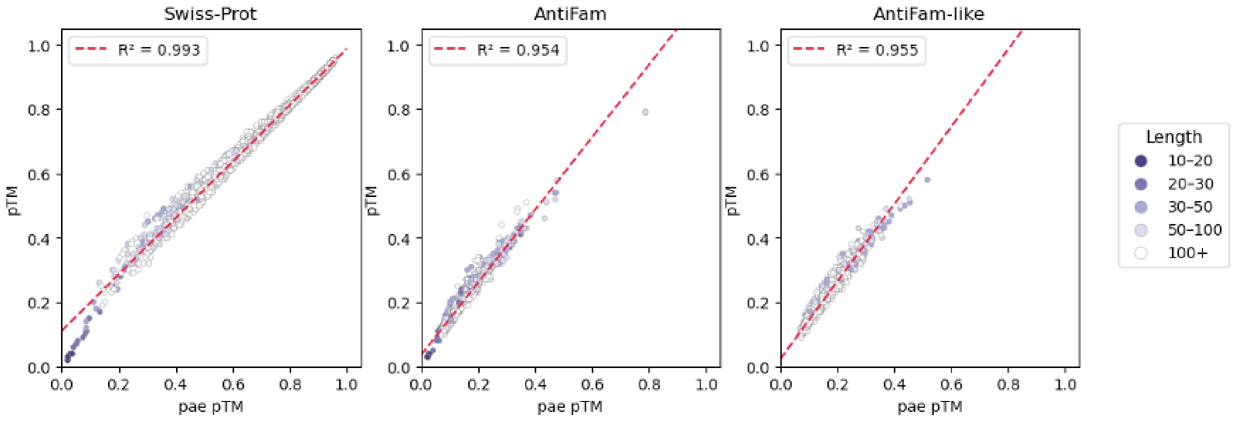

Supplementary Figure 1. Correlation between PAE-derived pTM and pTM scores for AlphaFold2 structure predictions of Swiss-Prot, AntiFam, and AntiFam-like sequences. Points are coloured by sequence length. Dashed lines indicate linear regression fits with R^2 values shown. Both scores are highly correlated ($R^2 > 0.95$) across all datasets.

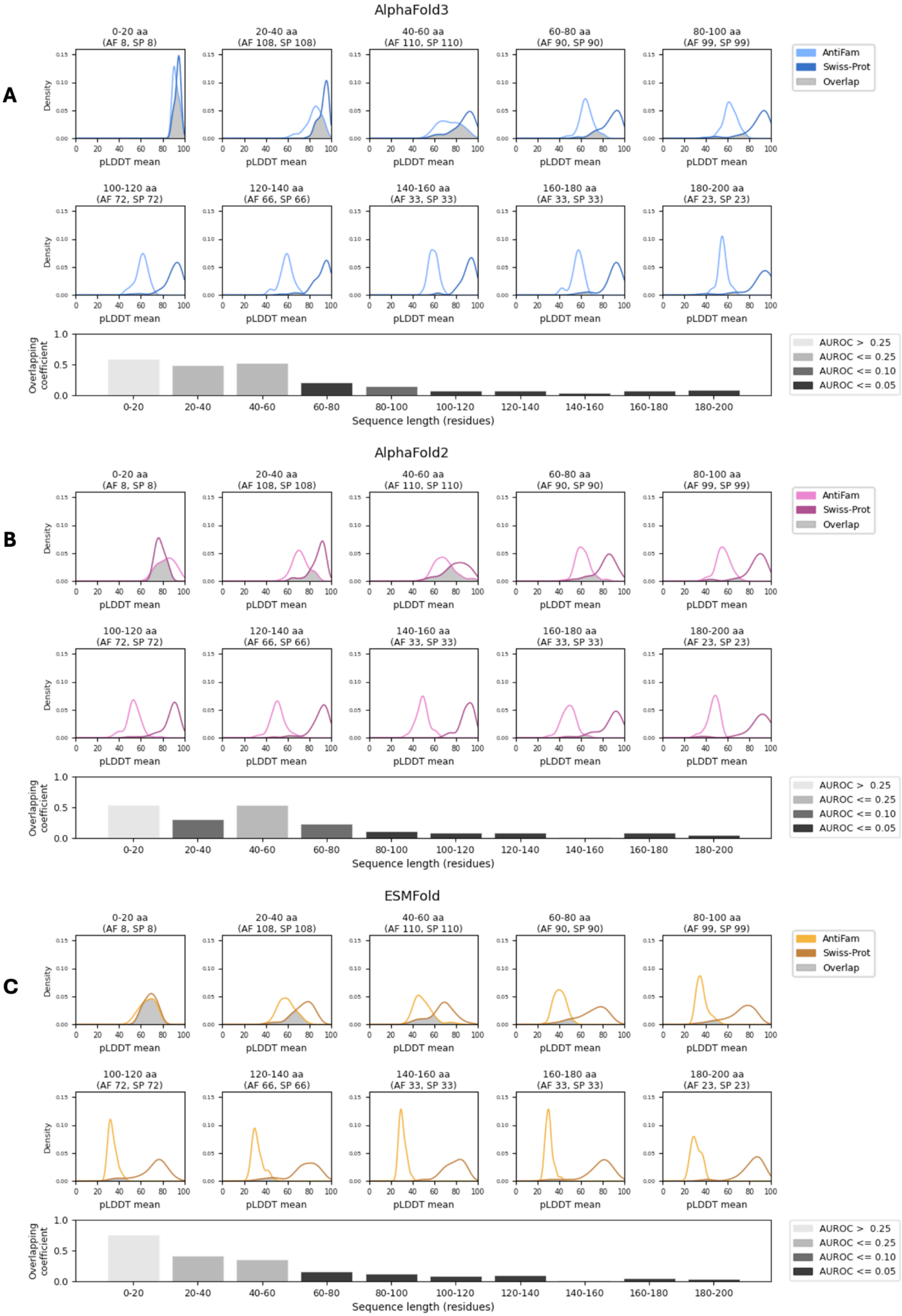

A
AlphaFold3
0-20 aa (AF 8, SP 8)
20-40 aa (AF 108, SP 108)
40-60 aa (AF 110, SP 110)
60-80 aa (AF 90, SP 90)
80-100 aa (AF 99, SP 99)
100-120 aa (AF 72, SP 72)
120-140 aa (AF 66, SP 66)
140-160 aa (AF 33, SP 33)
160-180 aa (AF 33, SP 33)
180-200 aa (AF 23, SP 23)
AntiFam
Swiss-Prot
Overlap
Density
pLDDT mean
Overlapping coefficient
Sequence length (residues)
AUROC > 0.25
AUROC <= 0.25
AUROC <= 0.10
AUROC <= 0.05
B
AlphaFold2
C
ESMFold

Supplementary figure 2. Overlapping Coefficient of pLDDT score distributions between Swiss-Prot and AntiFam, by sequence length bin (A), AlphaFold3, (B) AlphaFold2, (C) ESMFold. For each structure prediction method, *(Top)* Each panel shows the kernel density estimate (KDE) of pLDDT mean scores for Swiss-Prot (dark pink) and AntiFam (light pink) within a single length bin. The grey shaded region represents the area of overlap between the two distributions. *(Bottom)* Bar chart of the OC per bin, coloured by AUC.

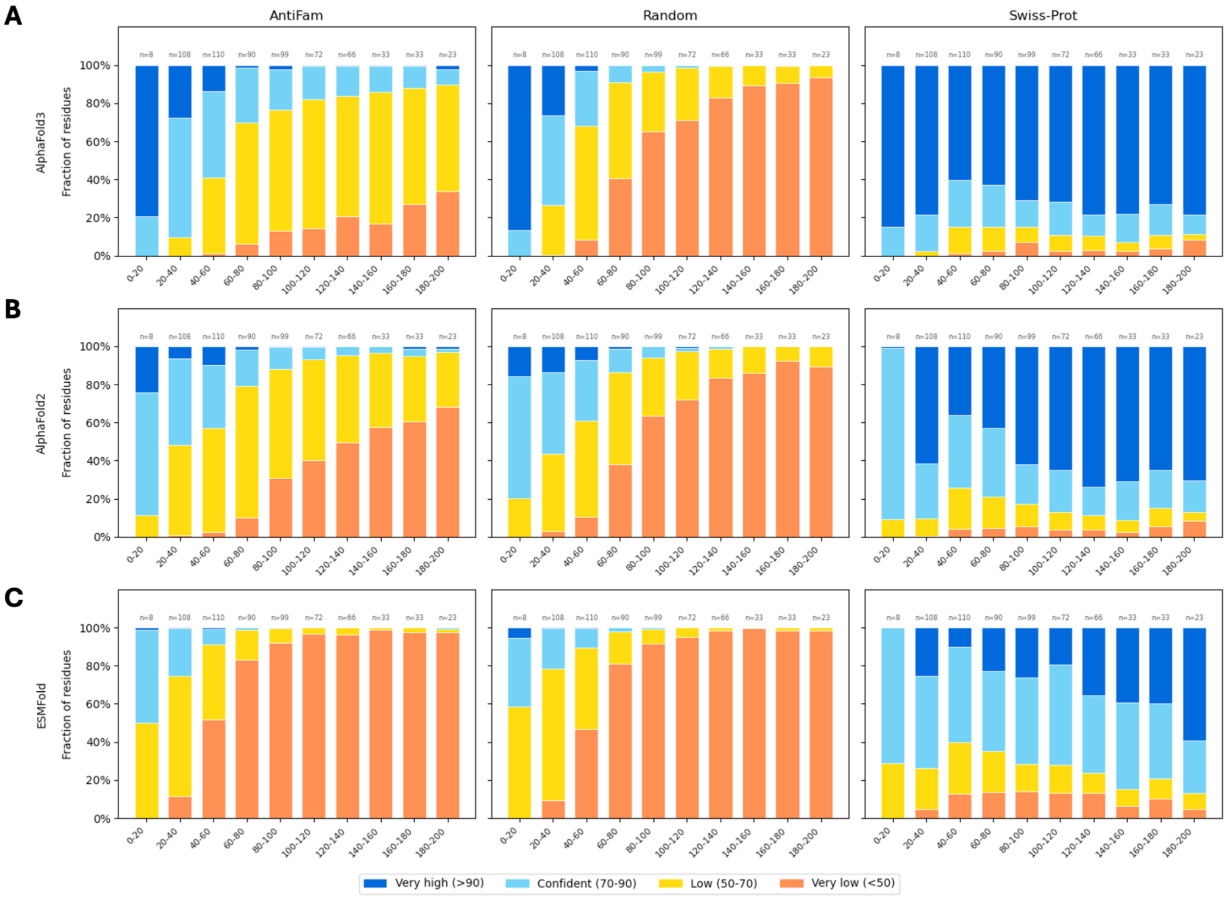

Supplementary figure 3. Distribution of pLDDT across sequence types AntiFam, Random, Swiss-Prot, averaged per sequence bins ranging (0-200), intervals of 20 per model type (A) AlphaFold3, (B) AlphaFold2), and (C) ESMFold.

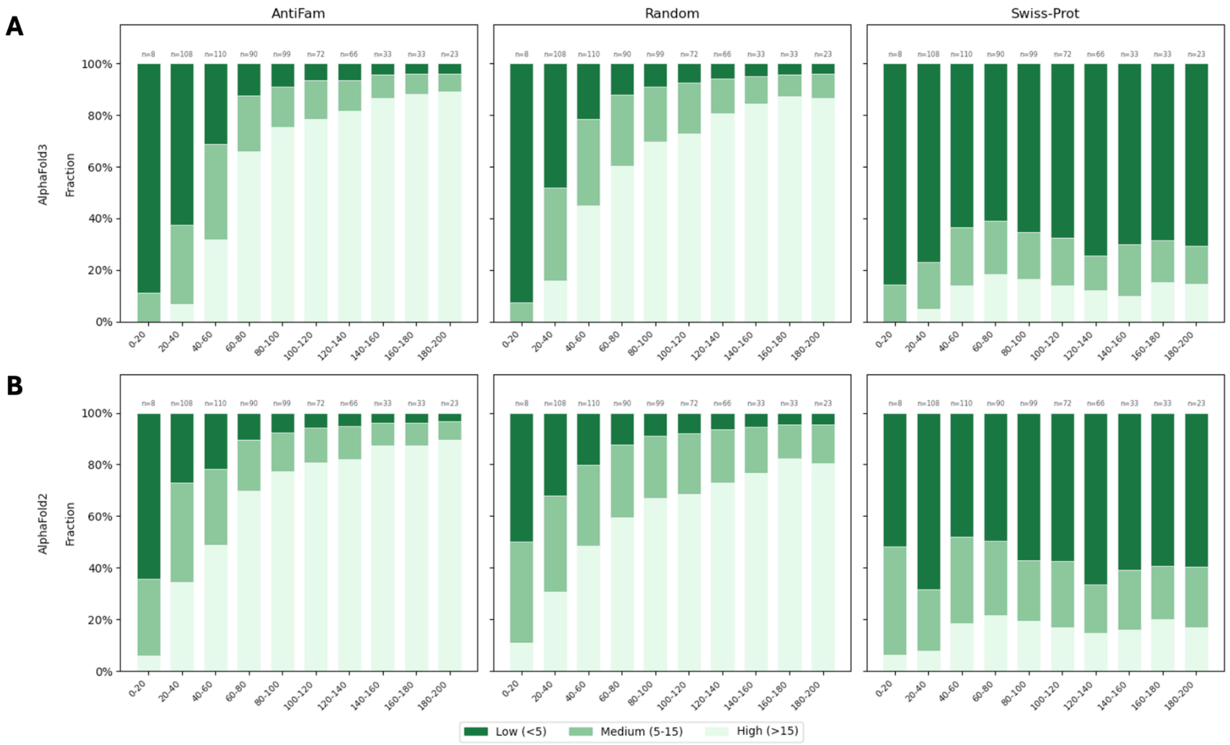

Supplementary figure 4. Distribution of predicted aligned error (PAE) across sequence types AntiFam, Random, Swiss-Prot, averaged per sequence bins ranging (0-200), intervals of 20 per model type (A) AlphaFold3 and (B) AlphaFold2.

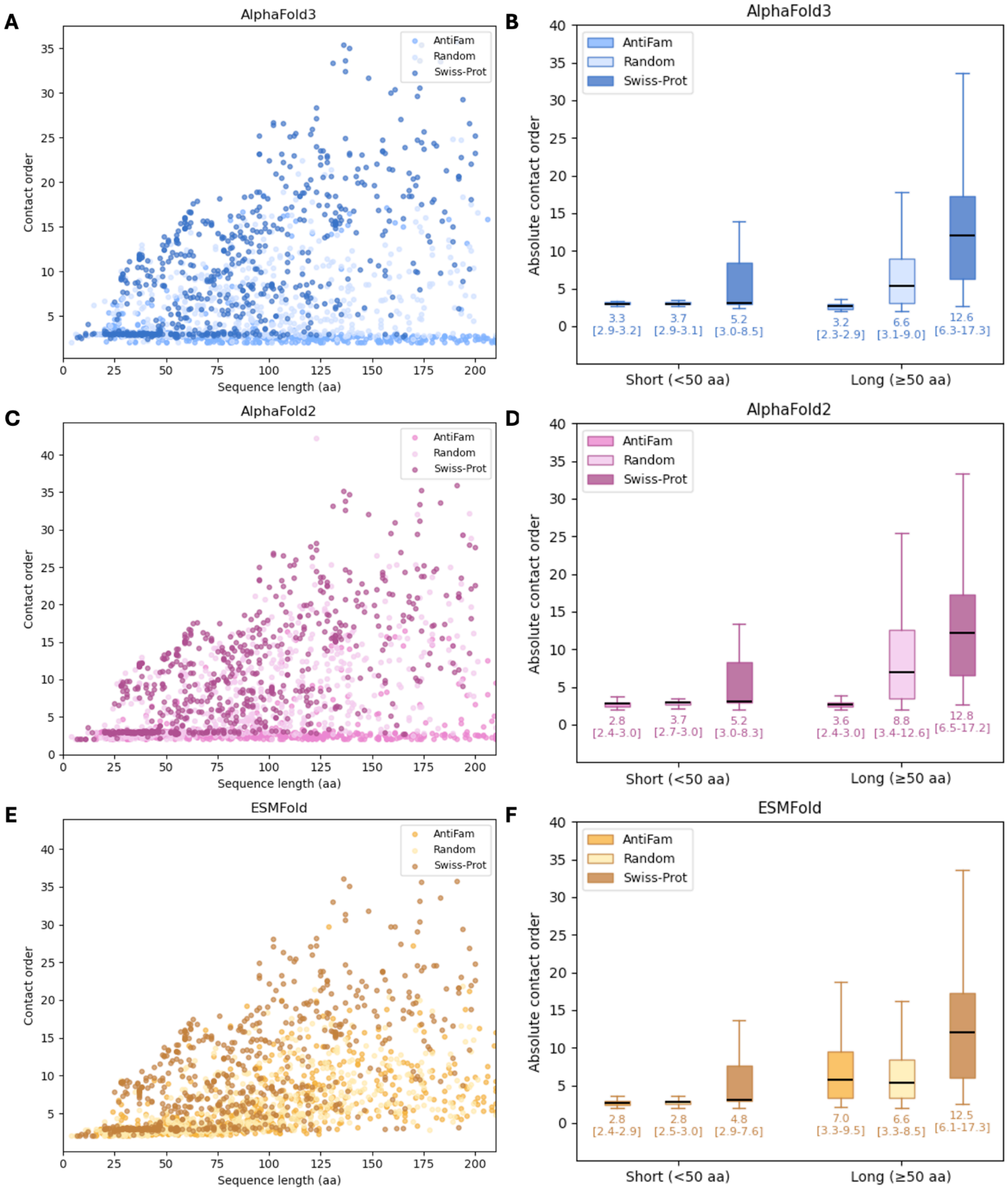

Supplementary figure 5. Contact order of AlphaFold3 (A), AlphaFold2 (B), and ESMFold (C) predicted structures across sequence types plotted against sequence length. Contact order measures the mean sequence separation of contacting residue pairs, where higher contact order indicates a greater proportion of contacts formed between residues far apart in the protein sequence, indicating more complex folded topologies and lower contact order indicates local, sequential contacts. Contacts are defined as Cα atom pairs within 8Å with a minimum sequence separation of ≥2 residues.

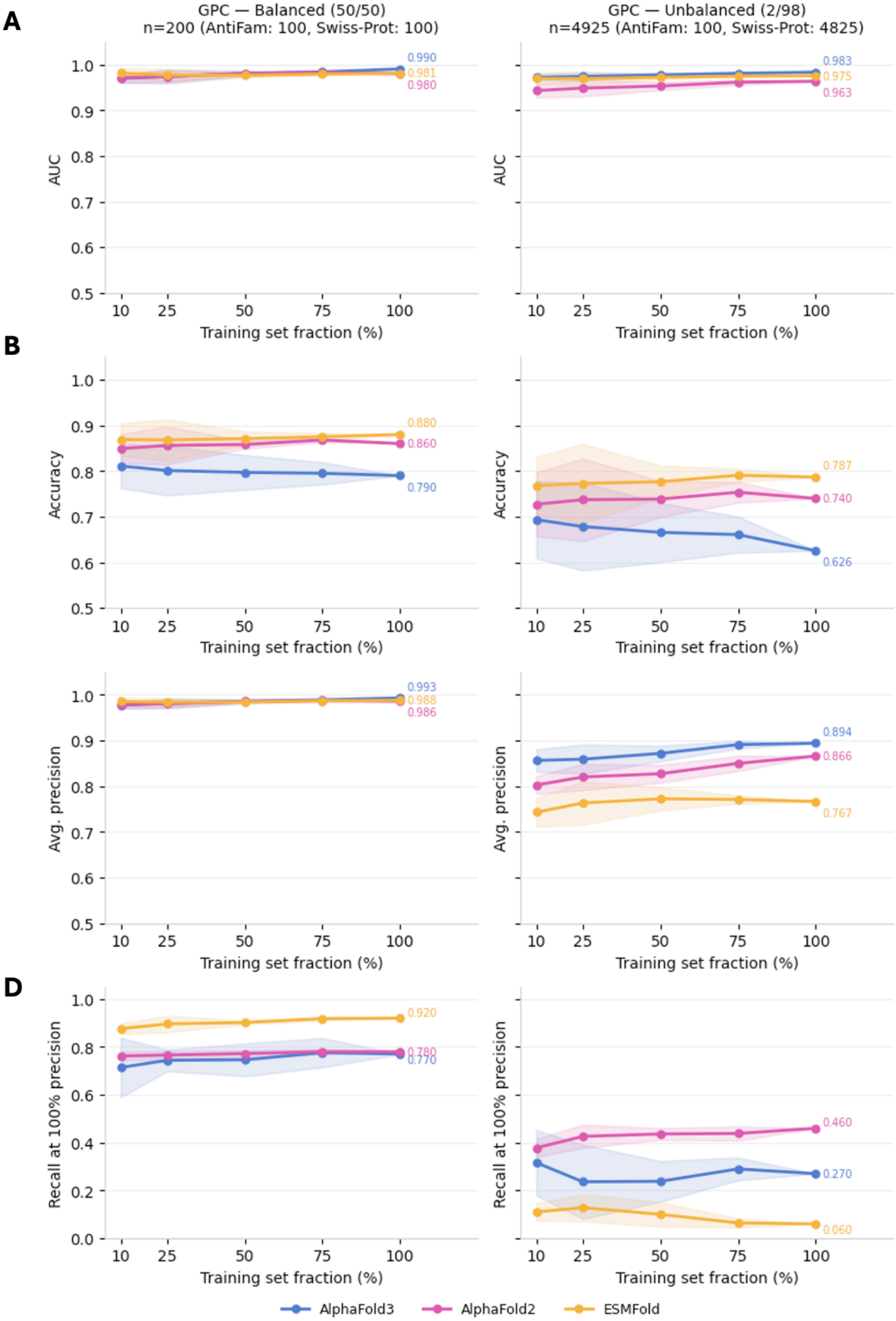
A
GPC — Balanced (50/50)
n=200 (AntiFam: 100, Swiss-Prot: 100)
GPC — Unbalanced (2/98)
n=4925 (AntiFam: 100, Swiss-Prot: 4825)
AUC
0.990
0.981
0.980
0.983
0.975
0.963
B
Accuracy
0.880
0.860
0.790
0.787
0.740
0.626
Avg. precision
0.993
0.988
0.986
0.894
0.866
0.767
D
Recall at 100% precision
0.920
0.780
0.770
0.460
0.270
0.060
Training set fraction (%)
AlphaFold3
AlphaFold2
ESMFold

Supplementary figure 6. GPC classification performance across varying training set sizes, five fractions ranging 10-100%, with five random seeds per training set fraction. Lines show mean performance for each model, and shaded bands show ±1 SD. GPC models were evaluated on a balanced test set (50% AntiFam-like / 50% Swiss-Prot, left column) and an unbalanced test set (2% AntiFam-like / 98% Swiss-Prot, right column). Four metrics are shown per row: AUC (area under the ROC curve), accuracy (proportion of correctly classified sequences at a decision threshold of 0.5), average precision (area under the precision-recall curve), and recall at 100% precision (maximum recall achievable at perfect precision).

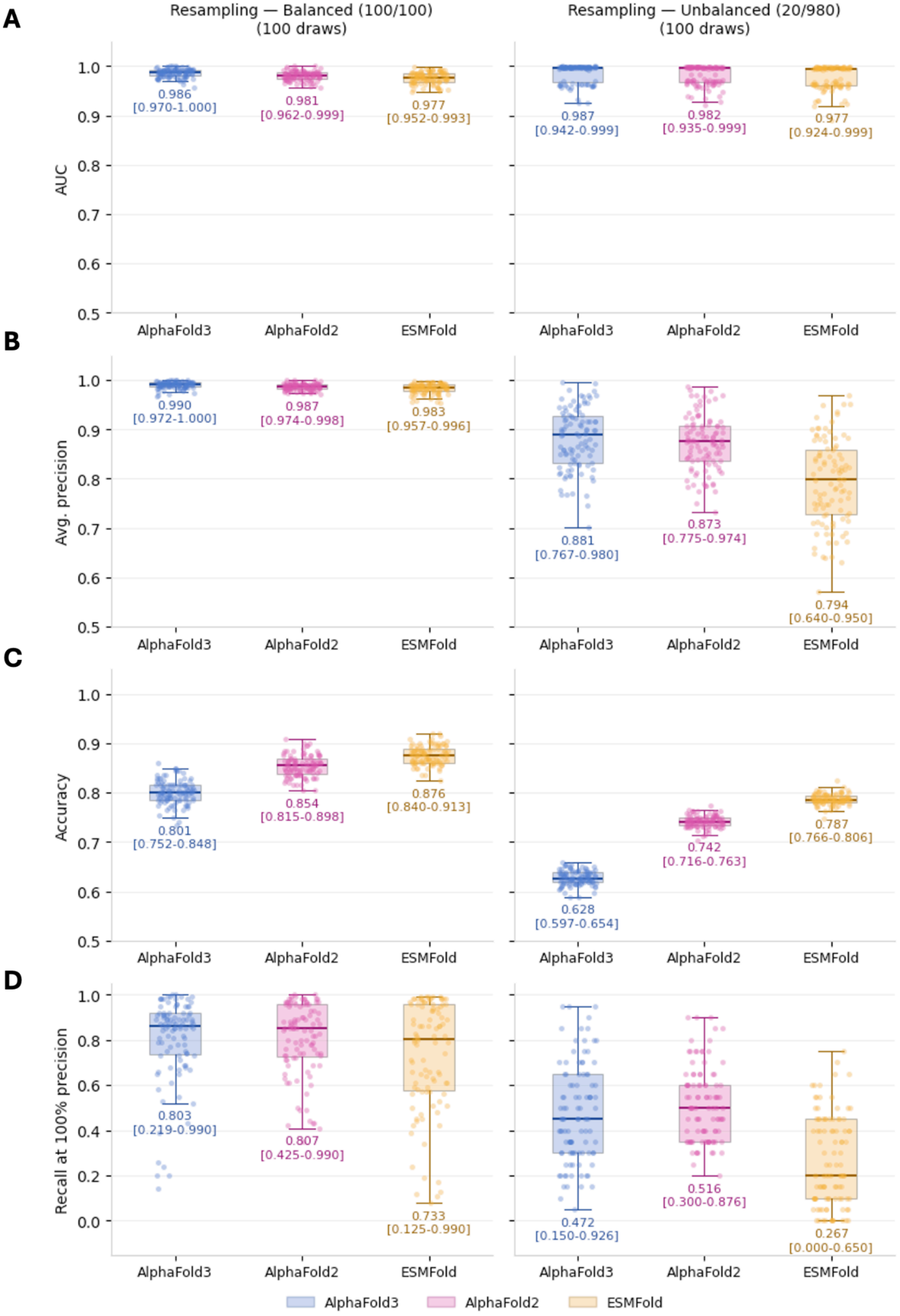

A
B
C
D
Resampling — Balanced (100/100) (100 draws)
Resampling — Unbalanced (20/980) (100 draws)
AUC
Avg. precision
Accuracy
Recall at 100% precision
AlphaFold3
AlphaFold2
ESMFold
0.986 [0.970-1.000]
0.981 [0.962-0.999]
0.977 [0.952-0.993]
0.987 [0.942-0.999]
0.982 [0.935-0.999]
0.977 [0.924-0.999]
0.990 [0.972-1.000]
0.987 [0.974-0.998]
0.983 [0.957-0.996]
0.881 [0.767-0.980]
0.873 [0.775-0.974]
0.794 [0.640-0.950]
0.801 [0.752-0.848]
0.854 [0.815-0.898]
0.876 [0.840-0.913]
0.628 [0.597-0.654]
0.742 [0.716-0.763]
0.787 [0.766-0.806]
0.803 [0.219-0.990]
0.807 [0.425-0.990]
0.733 [0.125-0.990]
0.472 [0.150-0.926]
0.516 [0.300-0.876]
0.267 [0.000-0.650]

Supplementary figure 7. Bootstrap resampling evaluation of GPCs. 100 test sets drawn per balanced (100 AntiFam-like/ 100 Swiss-Prot) and unbalanced (20 AntiFam-like / 980 Swiss-Prot) compositions, with proteins per draw consistent across GPC models. Jittered points show individual draws and boxes span IQR with whiskers extending to 1.5x ISQ. Mean and 95th percentile interval shown below whiskers. AUC calculated as area under ROC curve. Avg. precision calculated as area under precision-recall curve. Accuracy is the proportion of correctly classified sequences out of all sequences in the test set, computed at a fixed decision threshold of 0.5. Recall at 100% precision, is the maximum recall at perfect precision.
